# Hémosidérose du système nerveux central et son association avec un macroadénome hypophysaire: rapport de cas

**DOI:** 10.11604/pamj.2020.37.288.17507

**Published:** 2020-12-01

**Authors:** Jennifer Nyangui Mapaga, Mikel Martinez

**Affiliations:** 1 Service de Neurologie du Centre Hospitalier de Dax, Dax, France

**Keywords:** Hémosidérose, système nerveux central, adénome hypophysaire, Hemosiderosis, central nervous system, pituitary adenoma

## Abstract

Nous rapportons le cas clinique d´un patient de 58 ans qui présentait des céphalées depuis 3 mois dont les investigations paracliniques ont permis de poser le diagnostic d´hémosidérose du système nerveux centrale et son association rare avec un macro-adénome hypophysaire.

## Abstract (en)

We here report the clinical case of a 58-year-old patient with a 3-month history of headaches. Paraclinical investigations led to the diagnosis of hemosiderosis of the central nervous system showing a rare association with pituitary macroadenoma.

## Introduction

L´hémosidérose du système nerveux cérébrale est une entité clinique et radiologique rare, seulement une centaine de cas décrits dans la littérature [1]. L´hémosidérose du système nerveux centrale encore appelée hémosidérose superficielle ou encore sidérose marginale [2,3] fait référence à la décoloration brun rouille du système nerveux central secondaire au dépôt d´hémosidérine [4,5]. Sur le plan clinique une triade est classiquement rapportée. L´imagerie par résonance magnétique (IRM) reste l´examen de choix, la prise en charge repose sur la suppression de la cause du saignement, dans la forme idiopathique des traitements médicamenteux sont proposés dont l'efficacité reste inconstante.

## Patient et observation

**Figure 1:**
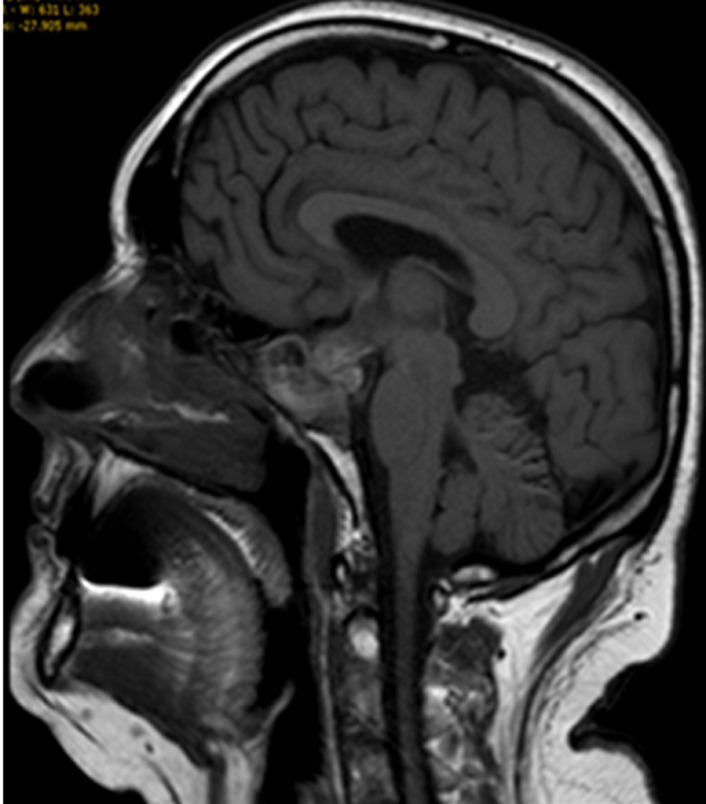
IRM cérébrale lésion d´aspect nodulaire développé à la face profonde de l´hypophyse, hypodense en T1

**Figure 2:**
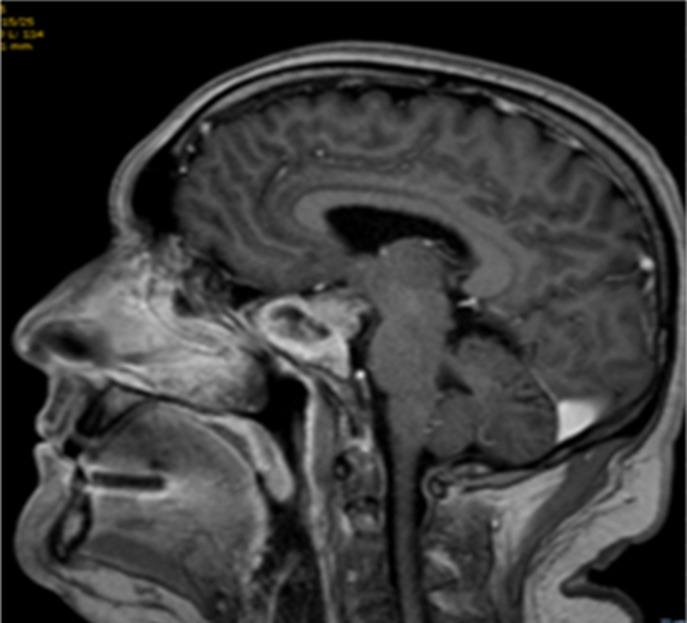
lésion hypodense au niveau de l´hypophyse prenant le contraste en T1 gado, la partie antérieure de la masse montre un aspect de nécrose

**Figure 3:**
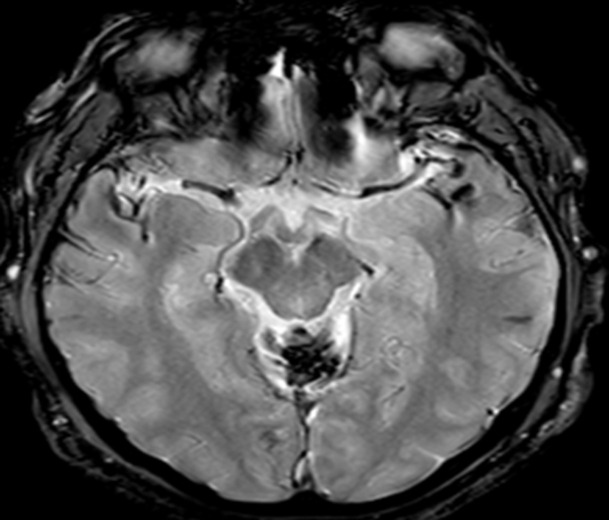
dépôts d´hémosidérines au niveau du vermis cérébelleux à l´IRM en séquence T2*

**Figure 4:**
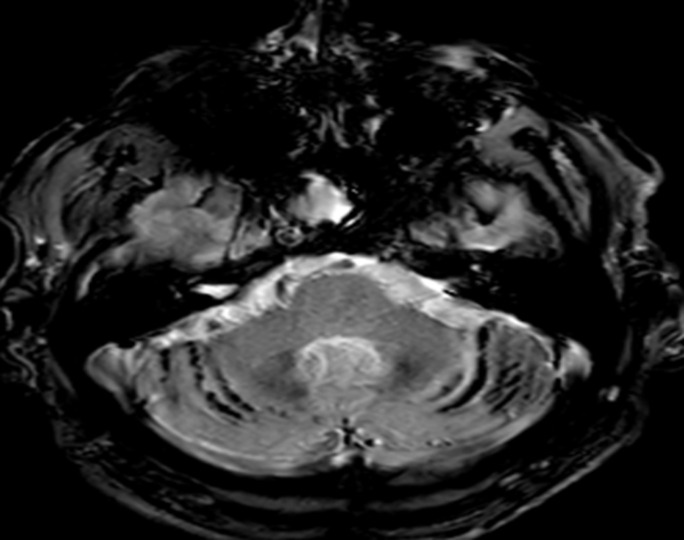
dépôts d´hémosidérines au niveau des hémisphères cérébelleux

**Figure 5:**
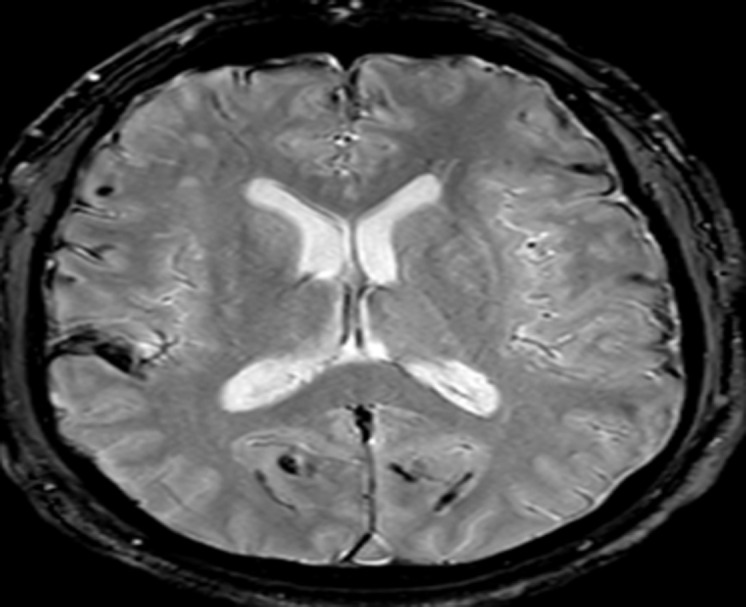
dépôts d´hémosidérines au niveau corticale à l´IRM cérébrale en séquence T2*

**Figure 6:**
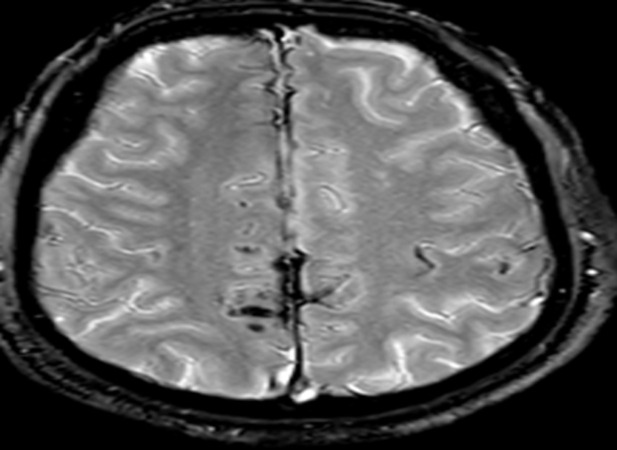
dépôts d´hémosidérines au niveau inter hémisphérique à l´IRM cérébrale en séquence en T2*

Nous présentons l´observation d´un patient âgé de 58 ans qui consulte pour des céphalées et une impression de voile devant les yeux depuis trois mois, il ne rapporte pas de troubles olfactifs ou auditifs. L'examen clinique retrouve une hémianopsie bitemporale, les fonctions cognitives sont conservées, on ne note pas de déficit sensitivomoteur ni de trouble de la coordination, les réflexes ostéo-tendineux normaux et cutanés plantaires en flexion, l´examen des autres appareils sont sans particularité. L´IRM cérébrale réalisée met en évidence une masse développée aux dépens de la selle turcique d´une taille d'environ 20 x 20 mm dans le plan coronal et sagittale en faveur d´un macroadénome hypophysaire (Figure 1, Figure 2) et le bilan biologique retrouve une hyperprolactidémie supérieur à 35 ng/ml. Un avis neurochirurgical a été demandé qui contre indique une opération devant la taille de l´adénome. Le patient est traité par Dostinex 1 comprimé mercredi et dimanche, Androtardyl 250 mg une injection tous les 21 jours. Le contrôle à 3 mois met en évidence une diminution de l´adénome et la présence de dépôt d´hémosidérine diffus en sus et sous tentoriel en séquence T2* (Figure 3, Figure 4, Figure 5, Figure 6), ces dépôts étaient déjà visibles dans la première IRM mais moins prononcés. Angio-IRM cérébrale ne montre pas de malformations vasculaires associées. L´IRM médullaire complémentaire ne retrouve pas de dépôts d´hémosidérose intramédullaire. La prise en charge a consisté à la surveillance imagerique et biologique tous les 6 mois. Le patient est stable actuellement l´hémosidérose persiste et la taille de l´adénome a diminué.

## Discussion

L´hémosidérose cérébrale est une pathologie rare, liée à un saignement chronique dans les espaces sous-arachnoïdiens entrainant des dépôts d´hémosidérine à la surface du cerveau, des nerfs crâniens et de la moelle épinière [6]. La physiopathologie, de cette affection n´est pas clairement établie, elle repose sur des hypothèses formulées par l´équipe d´A.H. Koeppen en 2008 qui montre qu´il existe 5 étapes pour aboutir à la sidérose du SNC. Les saignements sous-arachnoïdiens répétés permettent la libération d'hémoglobine, précurseur de l'hémosidérine. Plusieurs phénomènes cellulaires et biochimiques permettent cette conversion: l'hémoglobine présente dans le liquide cérébro-spinal (LCS) est transformée en hème. L'hème est ensuite transformé en fer puis en ferritine, elle-même transformée en hémosidérine, stockées dans les tissus et notamment dans le tissu glial cérébelleux [7]. L'incidence de la sidérose est estimée à 0,15% et elle est le plus souvent asymptomatique [8]. Quinze pourcents des patients présentent des manifestations cliniques à type surdité de perception unilatérale ou bilatérale dans 95% des cas, une ataxie cérébelleuse et un syndrome pyramidal. D´autres manifestations sont possibles notamment des paralysies de divers nerfs crâniens (paralysie oculomotrice, anosmie), une atteinte des fonctions supérieures et des céphalées comme observé chez notre patient, ces céphalées sont le plus souvent décrits dans la littérature comme étant des céphalées mises sur le compte d´hémorragies méningées, mais certaines peuvent également ressembler dans leur présentation à des céphalées migraineuses. D´autres sont chroniques et peuvent, soit être en rapport avec l´étiologie de la sidérose soit une complication de la maladie comme l´hydrocéphalie occasionnant des céphalées d´hypertension intracrânienne [9,10]. Les causes sont multiples mais retrouvées uniquement dans 50% des cas: tumeur cérébrale (épendymome, oligodendrogliome, astrocytome, méningiome) les tumeurs cérébrales sont responsables de 15% de tous les cas signalés de sidérose superficielle [11], malformation vasculaire, hemispherectomies, hématome sous dural, post-traumatisme crânien, avulsion radiculaire, angiopathie amyloïde dans 60% des cas. Toutes les lésions susceptibles de s'accompagner d'hémorragies sous-arachnoïdiennes chroniques ou répétées sont des étiologies potentielles [1,2].

L´IRM cérébrale et médullaire permet de poser le diagnostic avec une excellente sensibilité. Les dépôts d´hémosidérine apparaissent sous la forme d´hyposignaux sur les séquences T2 en écho de gradient, qui sont les séquences de choix pour porter le diagnostic. Ces anomalies siègent préférentiellement au niveau de la fosse postérieur: tronc cérébral, du cervelet, en particulier dans la portion haute du vermis et la partie antérieure des hémisphères, de la lame quadrigéminale, le long des portions initiales de la VIIIe paire crânienne, mais aussi autour de la moelle épinière ou le signal hypointense périmédullaire, en T2 écho de gradient, donne une fausse image de bande hyperintense centromédullaire. Les structures sus-tentorielles, sont moins fréquemment touchées et concernent les vallées sylviennes, la partie antéro-inférieure de la citerne interhémisphérique, les faces médiales des lobes temporaux, frontaux et occipitaux. La ponction lombaire constitue le deuxième examen à privilégier après l´imagerie par résonance magnétique. Le LCR est pathologique dans 75% des cas. Un LCR dépourvu d´anomalie ne doit donc pas remettre en cause le diagnostic, il est typiquement hémorragique et/ou xanthochromique, et il peut contenir des erythrophages ou des sidérophage. Un taux élevé de fer et/ou de ferritine peut être retrouvé, sans être spécifique de la maladie. La tomodensitométrie n'est pas indiquée, car elle ne montre le plus souvent que des signes indirects (atrophie cérébelleuse, en particulier). Le traitement de l'hémosidérose cérébrale repose sur celui de la cause supposée lorsqu'elle est connue et ne ralentira probablement que la progression du processus pathologique et la détérioration clinique [1,12]. Le traitement chirurgical de la sidérose superficiel dépend de l'identification précoce de la source du saignement. L'excision chirurgicale de la lésion incriminée (néoplasme ou malformation vasculaire ou pseudoméningocèles) et la réparation des défects duraux sont des stratégies thérapeutiques logiques. En cas de négativité du bilan étiologique, des traitements à base de chélateurs du fer ont été proposés, mais leur efficacité reste hypothétique [1,13-15].

## Conclusion

La sidérose superficielle est une affection rare, possiblement sous-estimé avant l´avènement de l´IRM. Le diagnostic doit être précoce pour retarder la progression de la maladie. Elle doit donc être connue des praticiens pour espérer des solutions de prise en charge face à cette affection.
